# The role of comprehensive geriatric assessment in older patients affected by knee osteoarthritis: an exploratory randomized controlled trial

**DOI:** 10.1007/s40520-025-03061-0

**Published:** 2025-05-16

**Authors:** Nicola Veronese, Anna Fazzari, Eleonora Santangelo, Candela Iommi, Pinar Soysal, Carlo Custodero, Lena Pickert, Maria Cristina Polidori, Nicoleta Stolniceanu, Helena Michalkova, Eva Topinkova, Alberto Pilotto, Mario Barbagallo

**Affiliations:** 1 Unit of Geriatrics, Department of Internal Medicine and Geriatrics, University of Palermo, Palermo, Italy; 2 Faculty of Medicine, Saint Camillus International University of Health Sciences, Rome, Italy; 3 Department of Geriatric Medicine, Faculty of Medicine, Bezmialem Vakif University, Adnan Menderes Bulvarı Vatan Street, 34093 Fatih, Istanbul, Turkey; 4 Department of Precision and Regenerative Medicine and Ionian Area (DiMePrev-J), Clinica Medica “Augusto Murri”, University of Bari Aldo Moro, Bari, Italy; 5 Aging Clinical Research, Department II of Internal Medicine, Center for Molecular Medicine Cologne, Faculty of Medicine, University of Cologne, University Hospital Cologne, Cologne, Germany; 6 Cologne Excellence Cluster on Cellular Stress Responses in Ageing-Associated Diseases (CECAD), University of Cologne, Cologne, Germany; 7 Department of Geriatrics and Internal Medicine, First Faculty of Medicine, Charles University, Prague, Czech Republic; 8 Faculty of Health and Social Sciences, University of South Bohemia, České Budějovice, Czech Republic; 9 Department of Interdisciplinary Medicine, University of Bari “Aldo Moro”, Bari, Italy; 10 Research, Development and Scientific Coordination Unit, National Reference, High Specialization Hospital, E.O. Galliera Hospitals, Genoa, Italy

**Keywords:** Osteoarthritis, Multidimensional, Comprehensive geriatric assessment, Pain, Disability

## Abstract

**Objectives:**

This study aimed to assess the effectiveness of Comprehensive Geriatric Assessment (CGA) compared to standard of care in improving pain, physical function, and stiffness in older adults with knee osteoarthritis (OA) over six months. Secondary outcomes included multidimensional frailty and quality of life.

**Design:**

An exploratory, multicentre, randomized controlled trial (RCT).

**Setting:**

Five European geriatric centres in Italy, Germany, Turkiye and the Czech Republic.

**Participants:**

Seventy older adults (mean age 76.1 ± 6.8 years; 80% female) with knee OA (Kellgren-Lawrence Grades 1–2) were randomized into two groups: CGA (*n* = 35) or standard of care (*n* = 35).

**Intervention:**

The CGA group underwent a multidimensional geriatric assessment and intervention, identifying impairments and tailoring interventions accordingly, while the control group received standard of care.

**Main outcome measures:**

The primary endpoint was improvement in pain, stiffness, and functional limitations measured using the Western Ontario and McMaster Universities Osteoarthritis Index (WOMAC) over six months. Secondary outcomes included changes in multidimensional frailty (Multidimensional Prognostic Index, MPI), quality of life (SF-36), and adherence to interventions.

**Results:**

The CGA group showed a non-significant improvement in total WOMAC scores (-4.49 ± 3.40, *p* = 0.19), with slight reductions in pain (-1.12 ± 0.96) and functional limitations (-3.26 ± 2.21). MPI slightly improved (-0.02 ± 0.04, *p* = 0.69), but no significant changes were observed in SF-36 scores. No falls, hospitalizations, or severe adverse events were reported.

**Conclusions:**

CGA may offer potential benefits for managing knee OA in older adults, particularly for pain and function, though statistical significance was not achieved. Larger studies with longer follow-up are warranted to confirm these findings.

**Trial registration:**

ClinicalTrials.gov Identifier: NCT05659979.

## Introduction

Osteoarthritis (OA) is the most common form of arthritis, and is characterized by joint pain and stiffness leading to functional decline and relevant loss in health related quality of life [1, 2]. The incidence of OA is rising due to the aging population and an increase in some risk factors [3], such as obesity. Knee OA is the most common OA localization, and symptomatic knee OA is highly prevalent among people aged over 50 years, affecting more than 250 million people worldwide [4].

Knee OA is a leading cause of pain in older people, and pain of the hip and knee results in physical disability and an increased risk of all-cause mortality [2, 5]. Hip and knee OA together are the eleventh highest contributor to global disability: the years of life lived with OA-related disability increased by 64% from 1990 to 2010 reaching 17 million [6]. OA is a progressive disorder, with different degrees of severity, that requires long-term management with various treatment options over the course of the disease [7]. The goals of treatment for OA are to reduce symptoms and ultimately slow disease progression, which may in turn reduce the impact of OA on the patient’s mobility and quality of life, with consequent reduction in healthcare resource needs.

In 2019, the European Society for Clinical and Economic Aspects of Osteoporosis, Osteoarthritis and Musculoskeletal Diseases (ESCEO) published recommendations for the management of knee OA in the form of a treatment algorithm that provides practical guidance for the prioritization of interventions and guides physicians through progressive, logical steps based on the severity of the knee OA signs/symptoms [8].

Currently, the management of knee OA is usually provided by several specialists, including general practitioners, rheumatologists, orthopedics and finally geriatricians. However, the exact role of geriatricians in the management of knee OA was poorly studied, whilst the comprehensive geriatric assessment (CGA) is widely used for preventing negative consequences in older people, such as hospitalization [9] or mortality [10]. Moreover, CGA can be used across different settings, from primary care to hospital, with similar beneficial effects in older people [11, 12]. Finally, people affected by knee OA are usually affected by other medical (e.g., dementia [13], cardiovascular diseases [14], depression [15]) and non-medical (e.g., loneliness [16]) conditions that can limit the adherence to therapeutic approaches, therefore limiting the efficacy of the interventions suggested in knee OA. To the best of our knowledge, no randomized controlled trials (RCTs) were conducted about CGA in older people affected by knee OA, even if CGA could be useful in people having knee OA, as shown by a systematic review of our group about this topic [17].

The primary aim of our investigation was to test the effectiveness of CGA, compared to standard of care, in improving pain, physical function, and stiffness in older people affected by knee OA over six months of follow-up. The secondary outcomes of our study included assessing the changes of multidimensional frailty and quality of life during the follow-up period as well as other negative outcomes such as hospitalization and mortality.

## Materials and methods

### Participants

This exploratory RCT was conducted in five different European centres (Bari, Cologne, Istanbul, Palermo, Prague) with a phase of enrolment between January 2023 and July 2024.

We considered, for the inclusion in this study, the following criteria: both genders; age > 60 years; able to sign the informed consent; diagnosis of knee OA according to standardized criteria in Grade 1 (doubtful narrowing of joint space and possible osteophytic lipping) and Grade 2 (definite osteophytes and possible narrowing of joint space) Kellgren and Lawrence system [18]. The only exclusion criterion was an estimated life expectancy less than 6 months.

The patients were enrolled among outpatients attending different ambulatories of geriatric services, such as cognitive disorders, osteoporosis, evaluation for disability or others. During a screening visit, their healthy condition was established by trained medical personnel on the strength of their medical history, a clinical examination, and routine biochemical tests.

The study was designed in accordance with the Helsinki Declaration and was approved by the Ethical Committee of the Azienda Ospedaliera Universitaria Policlinico Paolo Giaccone di Palermo (leading center) on 01st July 2022 and, then by Ethics Committees from all participating centres. All participants were fully informed about the nature, purpose, procedures, and risks of the study and gave their written informed consent. Our trial complied with the Consolidated Standards of Reporting Trials (CONSORT) statement for randomized trials [19]. The protocol was a priori registered in Clinical Trials (https://clinicaltrials.gov/study/NCT05659979).

### Randomization, intervention, and allocation

Patients were randomly assigned to one of the two groups, by using a computer-generated sequence of non-unique, unsorted numbers with a range from one to two representing the groups. The lists of randomizations were prepared before the enrollment by the Principal Investigator (PI) of the study (NV) and assigned to the Local PI, after the approval of the local Ethical Committee.

CGA group underwent to a standardized CGA by trained geriatricians that assessed the needs of older patients affected by knee OA in terms of disability, cognitive issues, social and nutritional problems, medications and comorbidities, and risk of pressure sores. All these domains were systematically assessed using the Multidimensional Prognostic Index (MPI) [20]. After the clinical assessment, the geriatricians proposed some tailored interventions for the domains impaired, including strategies for improving adherence to some background and/or first line medications, as detailed in the 2019 ESCEO algorithm [8]. The control group received a standard of care treatment.

The patients were followed up after the enrollment for six months, with one intermediate evaluation at three months in which we assessed the effect of CGA on primary and secondary outcomes compared to standard care.

### Outcomes

The primary endpoint of this study was to assess the efficacy of CGA compared to standard care in improving pain, physical function, stiffness over six months of follow-up using the Western Ontario and McMaster Universities Osteoarthritis Index (WOMAC) scale [21]. The WOMAC, that is one of the most used tool in knee OA research, measures five items for pain (score range 0–20), two for stiffness (score range 0–8), and 17 for functional limitation (score range 0–68) [21]. The single items of the WOMAC were assessed as co-primary outcomes.

For secondary outcomes, we included:

- A. The presence and severity of multidimensional frailty evaluated using the MPI [20] that includes:

(1) Functional status by Activities of Daily Living (ADL) index [22];
(2) Independence in the Instrumental Activities of Daily Living (IADL) [23];
(3) Cognitive status through the Short Portable Mental Status Questionnaire (SPMSQ) [24];
(4) Co-morbidity, in terms of presence and severity, examined using the Cumulative Illness Rating Scale (CIRS) [25] Severity Index (SI);
(5) Nutritional status investigated with the Mini Nutritional Assessment (MNA) short form (SF) [26];
(6) Risk of developing pressure sores was evaluated through the Exton Smith Scale (ESS) [27];
(7) Number of medications;
(8) Social domain was categorized in living alone, in family (or with other support) and in institution.

For each domain, a tripartite hierarchy was used, i.e. 0 = no problems, 0.5 = minor problems, and 1 = major problems, based on conventional cut-off points derived from the literature for the singular items [20]. The sum of the calculated scores from the eight domains was divided by 8 to obtain a final MPI risk score ranging from 0 = no risk to 1 = higher risk of mortality. MPI requires between 15 and 25 min for its complete execution [28].

b. B. Quality of life was assessed using the short-form 36 (SF-36) [29]. The SF-36 consists of eight scaled scores, which are the weighted sums of the questions in their section. Each scale is directly transformed into a 0-100 scale on the assumption that each question carries equal weight. We used the two aggregated scores, i.e., MCS (mental health) and PCS (physical health) [29]. The lower the score, the less quality of life.
c. C. Mortality was assessed using administrative data. Adverse events and severe adverse events, attributable to CGA or standard care, were reported in the CRFs of the patients and were recorded through interviews with the patients/relatives.

### Sample size calculation and statistical analysis

Based on a systematic review about the meaningful change in WOMAC score [30], for the sample size calculation for this exploratory RCT, we expected that the group allocated to CGA-treatment should improve, in mean, 8 points more than the group allocated to standard of care based on the primary outcome (WOMAC) at the main endpoint after six months of follow-up. With a common between-subject standard deviation of 14 points, sample size calculations show that 27 participants in each group are required to detect a statistical difference (power of 80% and significance level at 0.05 (two-sided). Therefore, a total of 60 participants were included, figuring out a drop-out rate of 10%.

All descriptive characteristics were reported using the means (with their standard deviations). Frequencies were reported as percentages in CGA and in control groups. Changes from baseline to six months on the primary and the secondary outcomes were analyzed using a repeated measure ANOVA (Analysis of Variance) test, including the change of all these outcomes at three months, comparing CGA to control group. An intention-to-treat analysis was proposed in this manuscript. The significance level was set at *p* = 0.05. Statistical analyses were performed using SPSS, version 26.0 software.

## Results

**Fig. 1:**
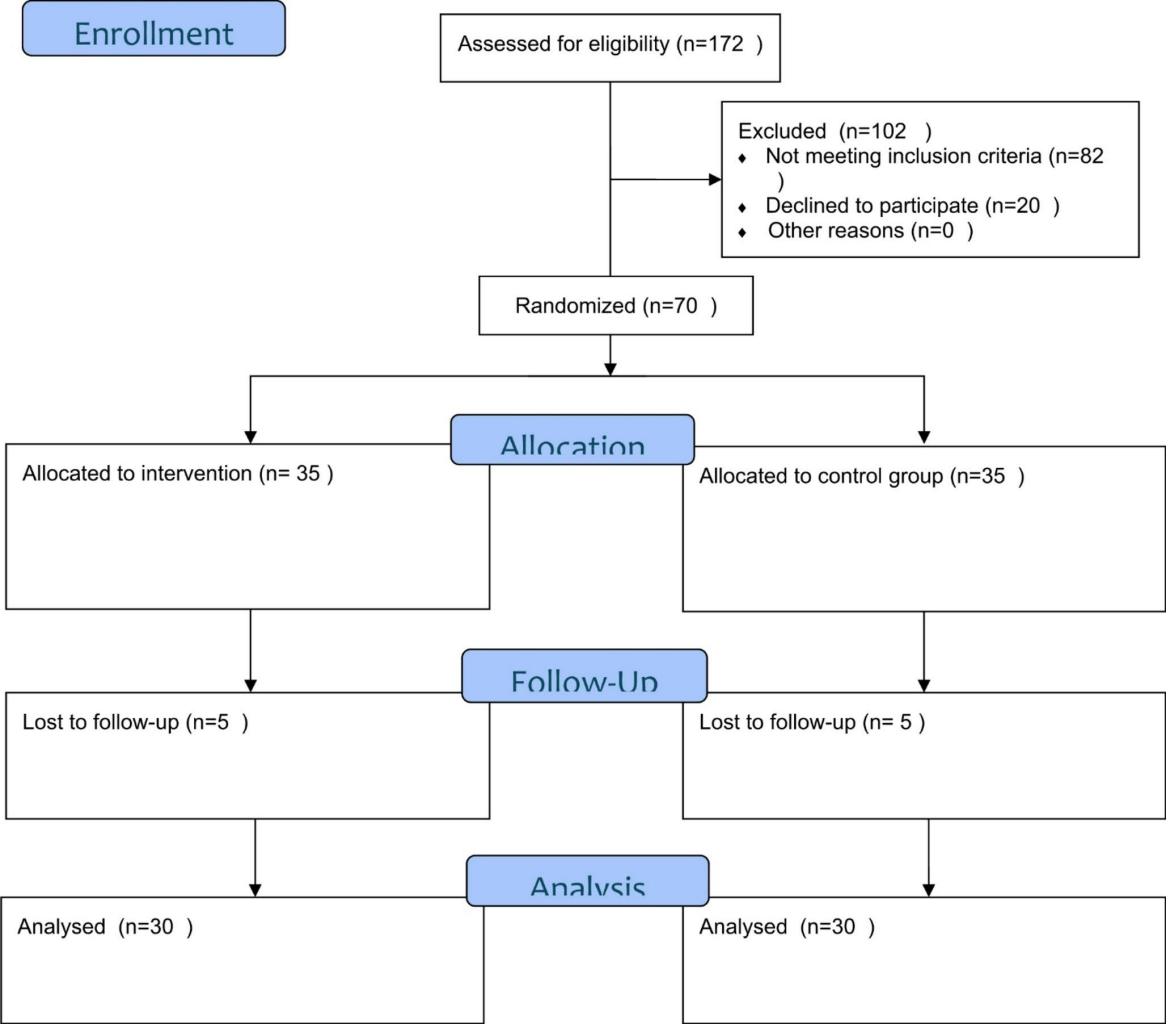
CONSORT 2010 flow diagram

Figure 1 shows the CONSORT flow diagram of our study. Among 172 patients initially assessed for eligibility, 102 were excluded mainly because they did not have a diagnosis of knee OA or a too severe form for our RCT. Finally, we randomized across the five European centers, 70 patients, allocating 35 patients to CGA and 35 to the control group.

Table 1 shows the descriptive characteristics of the patients included, divided by the kind of intervention proposed. Overall, the participants aged a mean of 76.1 years (SD = 6.8, range: 65–96) and they were mainly females (56/70, 80%). The participants experienced, in mean, a presence of moderate pain, stiffness and functional limitation according to the WOMAC subscales, having a total score of 40.2 points indicate moderate osteoarthritis symptoms. According to the multidimensional evaluation, fully reported in Table 1, we observed that the patients had minimal impairment in ADL or IADL and in nutritional aspects. Even if they scored low in CIRS-SI, they consumed, in mean, 6.4 medications. According to the MPI, 51.4% were ranked as robust from a multidimensional point of view (MPI < 0.33), 40.0% pre-frail (MPI 0.33–0.66), and 8.6% frail (MPI > 0.66).

**Table 1:** Descriptive characteristics of the participants

	CGA group (*n* = 35)	Control group (*n* = 35)
**Mean age**,** SD**	75.0 (5.8)	77.2 (7.5)
**Females (%)**	85.7	74.3
**WOMAC-pain subscale**	10.3 (4.3)	10.8 (4.3)
**WOMAC-stiffness subscale**	4.9 (2.1)	5.2 (1.8)
**WOMAC-functional limitation subscale**	25.5 (10.5)	24.3 (9.2)
**WOMAC-total**	40.2 (15.3)	40.3 (14.1)
**ADL**	4.5 (2.0)	4.9 (1.3)
**IADL**	5.4 (2.8)	5.6 (2.6)
**EES**	16.5 (2.4)	16.3 (2.1)
**SPMSQ**	1.2 (1.9)	1.5 (2.0)
**MNA-SF**	11.7 (2.2)	11.4 (2.4)
**Number of drugs**	6.7 (3.7)	6.0 (4.3)
**CIRS-SI**	0.41 (0.49)	0.36 (0.48)
**MPI**	0.39 (0.17)	0.36 (0.18)
**SF-36 MCS**	42.4 (11.6)	41.8 (11.2)
**SF-36 PCS**	45.6 (11.3)	44.3 (11.9)

**Abbreviations**: CGA – Comprehensive Geriatric Assessment; WOMAC – Western Ontario and McMaster Universities Osteoarthritis Index; ADL – Activities of Daily Living; IADL – Instrumental Activities of Daily Living; EES – Exton-Smith Scale; SPMSQ – Short Portable Mental Status Questionnaire; MNA-SF – Mini Nutritional Assessment – Short Form; CIRS-SI – Cumulative Illness Rating Scale Severity Index; MPI – Multidimensional Prognostic Index; SF-36

MCS–Mental Component Summary; PCS – Short Form-36 Physical Component Summary.

Data are reported as means and standard deviations or as percentages.

As shown in Table 2, During the six months’ follow-up, five participants for each group were lost, mainly due to a lack of interest in the study. About the primary outcomes, we observed that the participants randomized to CGA had an improvement in WOMAC score (mean difference=-4.49 ± 3.40 points), even if non-statistically significant (*p* = 0.19). Two WOMAC subscales were improved in CGA compared to control group (mean difference in pain: -1.12 ± 0.96; mean difference in functional limitations: -3.26 ± 2.21), even if these improvements were not statistically significant between the groups. About multidimensional evaluation, we observed a small reduction MPI in CGA compared to control group (mean difference: -0.02 ± 0.04; *p* = 0.69). No differences were observed about quality of life estimated with SF-36 MCS and PCS.

**Table 2:** Mean baseline characteristics and outcomes at follow-up, by group

	CGA group			Control group			Changes	
Parameter	Baseline	3 months	6 months	Baseline	3 months	6 months	Between-group change (6 months)	*p*-value
**WOMAC-pain subscale**	10.3 (4.3)	10.1 (4.2)	9.3 (4.5)	10.8 (4.3)	11.0 (4.4)	12.3 (3.9)	-1.12 (0.96)	0.23
**WOMAC-stiffness subscale**	4.5 (2.1)	4.9 (2.1)	4.7 (2.3)	5.2 (1.8)	5.0 (2.1)	5.4 (1.9)	-0.07 (0.47)	0.88
**WOMAC-functional limitation subscale**	25.5 (10.5)	23.0 (9.6)	23.1 (9.7)	24.3 (9.2)	26.2 (9.7)	28.2 (9.7)	-3.26 (2.21)	0.15
**WOMAC-total**	40.2 (15.3)	42.3 (14.9)	45.9 (14.2)	40.3 (14.1)	37.9 (15.0)	37.2 (15.7)	-4.49 (3.40)	0.19
**ADL**	4.5 (2.0)	4.6 (1.7)	4.5 (1.8)	4.9 (1.3)	5.0 (1.3)	5.0 (1.1)	-0.43 (0.35)	0.22
**IADL**	5.4 (2.8)	5.5 (2.5)	5.4 (2.4)	5.6 (2.6)	5.8 (2.3)	5.7 (2.0)	-0.24 (0.56)	0.66
**EES**	16.5 (2.4)	16.5 (2.2)	16.5 (2.3)	16.3 (2.1)	16.5 (2.0)	16.4 (2.1)	0.10 (0.50)	0.84
**SPMSQ**	1.2 (1.9)	1.1 (1.4)	1.2 (1.4)	1.5 (2.0)	1.4 (1.8)	1.5 (1.6)	-0.25 (0.35)	0.47
**MNA-SF**	11.7 (2.2)	11.8 (2.2)	11.7 (1.9)	11.4 (2.4)	11.7 (2.4)	11.9 (2.1)	0.06 (0.46)	0.90
**Number of drugs**	6.7 (3.7)	6.8 (3.9)	6.9 (3.9)	6.0 (4.3)	6.0 (4.3)	6.0 (3.9)	0.82 (1.06)	0.44
**CIRS-SI**	0.41 (0.49)	0.29 (0.45)	0.31 (0.45)	0.36 (0.48)	0.43 (0.50)	0.38 (0.46)	-0.10 (0.12)	0.43
**MPI**	0.39 (0.17)	0.37 (0.15)	0.36 (0.16)	0.36 (0.18)	0.37 (0.17)	0.38 (0.14)	-0.02 (0.04)	0.69
**SF-36 MCS**	42.4 (11.6)	41.4 (10.8)	42.1 (14.2)	41.8 (11.2)	42.1 (11.3)	41.9 (12.0)	0.03 (3.2)	0.84
**SF-36 PCS**	45.6 (11.3)	44.8 (10.1)	45.0 (11.2)	44.3 (11.9)	44.1 (12.3)	44.0 (12.0)	1.00 (3.1)	0.54

**Abbreviations: Abbreviations**: CGA – Comprehensive Geriatric Assessment; WOMAC – Western Ontario and McMaster Universities Osteoarthritis Index; ADL – Activities of Daily Living; IADL – Instrumental Activities of Daily Living; EES – Exton-Smith Scale; SPMSQ – Short Portable Mental Status Questionnaire; MNA-SF – Mini Nutritional Assessment – Short Form; CIRS-SI – Cumulative Illness Rating Scale Severity Index; MPI – Multidimensional Prognostic Index; SF-36

MCS–Mental Component Summary; PCS – Short Form-36 Physical Component Summary

Changes from baseline to six months on the primary and the secondary outcomes were analysed using a repeated measure ANOVA test, including the change of all these outcomes at three months, comparing CGA to control group

Finally, during the six months of follow-up, we did not detect any falls, mortality or hospitalization. No severe side effects attributable to the intervention were recorded.

## Discussion

The findings of this RCT suggest that CGA may provide some clinical benefits for older adults with knee OA, although the observed improvements did not reach statistical significance. Participants who underwent CGA demonstrated a mean improvement in the WOMAC score (-4.49 ± 3.40 points), with specific improvements in pain (-1.12 ± 0.96) and functional limitations (-3.26 ± 2.21), though these differences were not statistically significant. Additionally, there was a slight reduction in multidimensional impairment (MPI difference: -0.02 ± 0.04; *p* = 0.69), but no significant changes in quality of life as measured by the SF-36. These results suggest that while CGA may have a role in managing knee OA, further investigation is necessary to confirm its clinical efficacy.

CGA was effective in reducing several negative outcomes in older people. A recent umbrella review about CGA showed that, in RCTs, this kind of intervention reduces some important outcomes typical of older people, such as nursing home admission and pressure sores in hospital medical setting and the risk of delirium in hip fracture, supported by a high certainty of evidence [31]. However, all these findings mainly derive from acute conditions, whilst the evidence of CGA as intervention in the management of chronic conditions is still limited. One of the key clinical implications of the findings of our RCT is the potential for CGA to complement standard OA management among frail older people. While traditional OA treatment focuses on pharmacologic and surgical interventions, CGA may incorporate indications for physical therapy, nutritional guidance, mental health support, and social considerations [32]. At the same time, CGA could be important to reduce inappropriate medications [33] or to better tailor some pharmacological therapies, as proposed in the multimodal management of knee OA [34]. However, given the lack of statistically significant differences between the intervention and control groups, its role in delaying the need for invasive interventions such as total knee replacement remains uncertain. Additionally, the minimal impact on quality of life measures suggest that CGA alone may not be sufficient for improving overall well-being in this population.

Several limitations should be considered when interpreting these findings. First, the relatively small sample size (*n* = 70) may have limited the statistical power to detect significant differences. We calculated, in fact, that this sample size to have a difference between the two groups of 8 points in the WOMAC scale, while we observed a mean difference of approximately 4 points. The short follow-up period of six months may also have been insufficient to capture long-term benefits of CGA. Furthermore, a notable proportion of participants (10 in total) were lost to follow-up, potentially introducing bias, even if we used an intention-to-treat analysis. Additionally, adherence to CGA recommendations may have varied, influencing the effectiveness of the intervention. Future studies with larger sample sizes, extended follow-up periods, and strategies to enhance adherence are warranted to better evaluate the potential benefits of CGA: we believe that our exploratory study has also the importance to underline some methodological limitations in order to better organize the future RCTs about this important topic.

In conclusion, this study provides preliminary evidence suggesting that CGA may have some beneficial effects in the management of knee OA, particularly in pain and functional outcomes, though these did not reach statistical significance. Future trials should aim to clarify the long-term benefits, cost-effectiveness, and practical implementation strategies of CGA to determine its role in standard OA care. These findings contribute to the ongoing exploration of multidisciplinary approaches for managing chronic musculoskeletal conditions in geriatric populations.

## Data Availability

No datasets were generated or analysed during the current study.
